# Targeted Therapies: Friends or Foes for Patient’s NK Cell-Mediated Tumor Immune-Surveillance?

**DOI:** 10.3390/cancers12040774

**Published:** 2020-03-25

**Authors:** Laura Damele, Selene Ottonello, Maria Cristina Mingari, Gabriella Pietra, Chiara Vitale

**Affiliations:** 1 UO Immunologia IRCCS Ospedale Policlinico San Martino, 16132 Genoa, Italy; lauradamele91@gmail.com (L.D.); sele_8@hotmail.it (S.O.); mariacristina.mingari@unige.it (M.C.M.); gabriella.pietra@unige.it (G.P.); 2 Dipartimento Medicina Sperimentale (DIMES), Università degli Studi di Genova, 16132 Genoa, Italy; 3 Centre of Excellence for Biomedical Research (CEBR), Università degli Studi di Genova, 16132 Genoa, Italy

**Keywords:** NK cells, targeted immunotherapy, cancer immunosurveillance

## Abstract

In the last 20 years there has been a huge increase in the number of novel drugs for cancer treatment. Most of them exploit their ability to target specific oncogenic mutations in the tumors (targeted therapies–TT), while others target the immune-checkpoint inhibitor molecules (ICI) or the epigenetic DNA modifications. Among them, TT are the longest established drugs exploited against a wide spectrum of both solid and hematological tumors, often with reasonable costs and good efficacy as compared to other innovative therapies (i.e., ICI). Although they have greatly improved the treatment of cancer patients and their survival, patients often relapse or develop drug-resistance, leading to the impossibility to eradicate the disease. The outcome of TT has been often correlated with their ability to affect not only tumor cells, but also the repertoire of immune cells and their ability to interact with cancer cells. Thus, the possibility to create novel synergies among drugs an immunotherapy prompted scientists and physicians to deeply characterize the effects of TT on immune cells both by in-vitro and by ex-vivo analyses. In this context, NK cells may represent a key issue, since they have been shown to exert a potent anti-tumor activity, both against hematological malignancies and solid tumors. In the present review we will discuss most recent ex-vivo analyses that clarify the effect of TT treatment on patient’s NK cells comparing them with clinical outcome and previous in-vitro data.

## 1. Introduction 

Targeted therapies (TT) act by inhibiting and disrupting biochemical pathways that play a key role in tumor cell growth and survival. They have been shown to induce significative tumor regression and improve overall survival of molecularly defined subsets of patients, both in solid and hematological malignancies [1,2,3].

Imatinib was the first tyrosine kinase inhibitor (TKI) designed to interfere with kinase activity of p190 and p210 oncoproteins, generated by the chimeric gene BCR-ABL, originating from translocation between terminal fragment of chromosome 9 and chromosome 22, that generates the Philadelphia chromosome (Ph^+^), the typical marker of chronic myeloid leukemia (CML) [4]. The dramatic success of this drug opened the door to the identification of novel molecular targets and to the design on novel drugs. In solid tumors the main target of these drugs are oncoproteins crucial for tumor maintenance and survival such as epidermal growth factor (EGFR), B-Raf proto-oncogene/serine/threonine kinase (BRAF), proto-oncogene c-Kit (KIT), human epidermal growth factor receptor 2 (HER2) [3,5].

Patients generally respond rapidly to these small-molecule kinase inhibitors however, after striking initial regression, tumors develop drug-resistance variants, leading to the progression of the disease. This phenomenon is particularly relevant for solid tumors, while in hematological tumors another major issue is the life-long treatment that these therapies may require, as their discontinuation may lead to relapse in half of patients [5,6]. Thus, these therapies are not curative, may exert several sides effects and raise concerns for the economic burden of treatments [6,7]. Among off-sides effects, these compounds exert an immunomodulatory effect of the immune system due to their ability to target several proteins involved in immune system development and activation such as proto-oncogene stem cell factor receptor (c-Kit), platelet-derived growth factor receptor (PDGF-R) and vascular endothelial growth factor receptor (VEGF-R), proto-oncogene tyrosine-protein kinase Src kinase family and mitogen-activated protein kinases (MAPK) [8,9]. Thus, several studies attempted to clarify the off-sides effects of these compounds and their interactions with immune systems to define mechanisms contributing to the success of the treatment or to its failure. 

In the present review we will focus our attention on the effect of TT on human natural killer (NK) cells in vitro and in vivo. NK cells have been proved to play an important role in anti-tumor immune response, particularly in hematological malignancies. On the other hand, in solid tumors, the exploitation of these cytotoxic cells has resulted very difficult to fulfill up to now and several strategies have been proposed [10,11,12,13,14]. Thus, a detailed analysis of the effects of TT therapy may contribute to understand the possibilities to improve NK cell-mediated control of neoplastic disease. 

## 2. Natural Killer Cells

**Figure 1:**
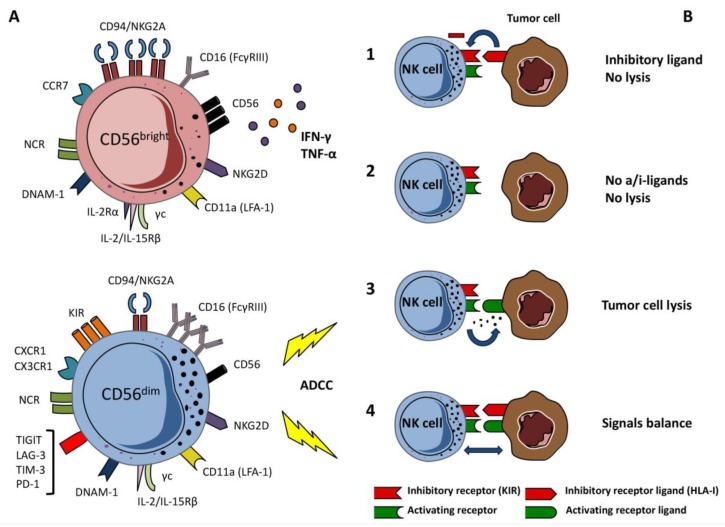
Natural Killer (NK) cells subsets and their functions. (**A**) NK cells subsets: CD56^dim^ cytotoxic NK subset express low levels of CD56 molecule and are able to perform antibody-dependent cellular cytotoxicity (ADCC) thanks to the high expression of CD16 receptor; CD56^bright^ NK subset express high levels of CD56 molecule and is capable to produce interferon-γ (IFN-γ) and TNF-α, thus contributing to the activation of monocytes and Th1 response. (**B**) NK cells exert cytotoxic activity and cytokines release through a balance between activating and inhibitory receptors: (**1**) The target cell cannot express ligands for activating receptor and express normal levels of Human Leukocyte Antigen class I molecules (HLA-I) blocking NK cell response; (**2**) In the absence of HLA class I molecules NK cell cytotoxicity can be inhibited by the lack of expression of activating receptor ligands on tumor cells; (**3**) NK cell activity is exerted by the binding of ligand to activating receptor and thanks to the lack of HLA-I molecules on target cell; (**4**) The normal expression of both ligands leads to a dynamic balance between NK cell and target cell.

NK cells are thought to represent one of the major host effector cells to control tumor development and progression [11,13,15,16]. Upon activation, they can exert powerful cytotoxicity against tumor- transformed cells by the release of molecules such as perforin and granzymes, of several chemokines such as MIP-1α/β, Chemokine (C-C motif) Ligand 5 (CCL5), Chemokine (C-X-C motif) Ligand 1 (CXCL1), and cytokines, such as Interferon (IFN)-γ, Tumor Necrosis Factor (TNF)-α, Granulocyte-Macrophage Colony-Stimulating Factor (GM-CSF), or Interleukin (IL)-10, which can differently modulate inflammatory responses [15,17,18]. NK cells represent 5–15% of circulating lymphocytes in peripheral blood (PB), and can migrate in peripheral tissues and in secondary lymphoid tissues (SLT) [19,20] (Figure 1). In SLT, they provide an early source of IFN-γ and interact with dendritic cells (DCs) playing an important role in DCs maturation and thus in the promotion T helper cell type 1 (Th1) responses [21,22,23].

The NK cell rapid and strong activation requires a tight control that is mediated by a large repertoire of inhibitory and activating receptors [24] (Figure 1). In humans, the main inhibitory molecules include a complex group of clonally distributed HLA-I-specific receptors, the killer Ig-like receptors (KIRs) and CD94/NKG2A: the C-type lectin CD94/NKG2A receptor binds HLA-E molecules sensing the global HLA-class I molecules on the target, while KIRs bind classical HLA-class I molecules, including HLA-C, HLA-Bw4, and some HLA-A alleles [25,26,27]. The inhibitory KIRs and the NK group 2 member A (NKG2A) receptor are also involved in NK cell education as they drive the functional maturation of NK cells expressing KIR specific for self HLA-class I molecules, inducing the acquisition of a strong cytolytic activity against pathological cells losing HLA class I expression [28].

Activating receptors include the natural cytotoxicity receptors (NCRs: NKp46, NKp30, NKp44), NKG2D, DNAX Accessory Molecule-1 (DNAM-1) and co-receptors such as 2B4 (CD244), NTBA (CD352) and NKp80 [29]. These receptors have the ability to recognize specific ligands on virus-infected cells or tumors [30,31] Ig-like receptor family includes NKp30, NKp44 and NKp46 and DNAM-1: only some ligands of these receptors have been identified, including BAT3/BAG6 and B7H6 (NKp30-L), mixed-lineage leukemia-5 (MLL5), sindecan-4, galectin-3 and soluble ligand NID1 (NKp44-L), CFP and other viral ligands (NKp46-L), PVR (CD112) and nectin-2 (CD155) are DNAM-1 ligands, while MIC-A/B and ULBPs are ligands for NKG2D receptor, which is a C- type lectin molecule [31,32,33]. The 2B4 receptor belongs to the family of signaling lymphocyte activation molecule (SLAM)-related receptors (SRR) and recognizes CD48 molecule. NK-T-B-antigen (NTB-A) protein is similar to 2B4 protein both structurally and functionally, but the activation requires a homophile bond with another NTB-A molecule [34]. Finally, NKp80 receptor, expressed as dimer, recognizes AICL molecule (expressed by activating monocytes and tumor cells) [35]. NK cells can also express other immunomodulatory receptors such as TIGIT (T cell immunoreceptor with Ig and ITIM domain), CD96, TIM-3 T cell immunoglobulin domain and mucindomain-3), PD-1 (programmed death-1) and LAG-3 [36]. TIGIT and CD96 are detectable on resting NK cells and compete with the activating receptor DNAM-1 for the same ligands, in particular for CD155. Thus, crosslinking of CD155 by TIGIT inhibits NK cell cytotoxicity, while CD96 seems to preferentially modulate IFN-γ production [37]. TIM-3 is expressed by resting CD56^dim^CD16^+^ NK cells while CD56^bright^ NK cells may acquire its expression upon cytokine stimulation. It is considered a marker of NK cell activation/maturation [38]: main ligands are galectin-9, phosphatidyl serine, high mobility group box-1 (HMGB-1) and carcinoembryonic antigen-related cell adhesion molecule-1 (CEACAM-1). TIM-3 inhibitory activity on NK cells is still controversial and seems to depend upon the type of stimulation used [36]. PD-1 is expressed on limited percentages of resting CD56^dim^CD16^+^ KIR^+^CD57^+^ NK cells. 

Similarly to TIM-3, it is considered a marker of NK cell terminal differentiation, identifying memory-like NK cells or functionally exhausted NK cells. Finally, LAG-3 is expressed on activated NK cells but its role on human NK cell functions is still unclear [36]. Generally, most these receptors are upregulated on human NK cells isolated from PB or tissue biopsies from patients affected by different types of tumors, suggesting that their acquisition favors the enrichment in NK cells with low functionality or exhausted features [36].

Receptor interaction with ligand leads to activation of NK cytolytic activity but, in the absence of strong cytokine activation, the engagement of at least two of activating receptors on NK cells is required to trigger degranulation [39,40]. On the other hand, the other major activating receptor, the Fc**γ**RIIIA receptor (CD16), can trigger degranulation alone upon binding of an antibody bound to a target cell, the so called antibody-dependent cellular cytotoxicity (ADCC) [40]. 

In addition, NK cells may express the activating counterparts of HLA-class I specific receptors (i.e., activating KIRs and CD94/NKG2C). The specificity of these latter receptors for HLA-I molecules has been formally defined only for KIRDS1, KIR2DS4 and NKG2C, and their role in addressing NK cytotoxicity has not yet been clearly defined [41,42,43,44,45,46].

The type and the quantity of receptors involved in NK-target cell interaction dictates the nature (activating vs inhibitory) of the immunological synapse and the consequent decision on granule release. Many tumor cell lines often express the ligands for different activating receptors and show reduced expression of HLA-class I molecules thus representing potential NK-sensitive targets in vivo (Figure 1).

NK cells are commonly clustered in two main subsets that allow them to patrol the body and interact with different immune cell types in different tissues (Figure 1). In particular, NK cell subsets are differently represented in PB and SLT, representing NK cells in sequential stages of differentiation with different functional features [20,47]. “Terminally differentiated” PB CD56^dim^CD16^bright^ NK cells expressing CD57 and KIR molecules display high cytotoxic potential and may release high amounts of IFN-γ upon NCR triggering. They represent the majority of circulating PB NK cells. On the other hand, the CD56^bright^CD16^dim^CD57^-^KIR^-^NKG2A^++^ express low perforin levels, are poorly cytotoxic but release large amounts of IFN-γ upon cytokine stimulation [48,49]. They are mainly distributed in SLT and several tissues. In PB, they can be considered the precursors of CD16^dim^ NK cells [19,20,47].

## 3. NK Cells and Hematological Malignancies 

NK cells have been shown to kill in vitro a number of established or primary cell lines derived from several hematologic malignancies, including T and B leukemias, myeloid leukemias, lymphomas, and multiple myelomas [30,35,50,51,52,53]. Most activating receptors, including the NCRs, NKG2D and DNAM-1 are involved in the recognition and killing of transformed cells [30]. NKG2D and DNAM-1 receptors as well as the NCRs have been reported as important for the targeting of acute and chronic myeloid leukemias (AML,CML) blasts, acute lymphoblastic leukemia (ALL) [30,51,52,54,55], multiple myeloma [53] while the role of DNAM-1 receptor seems to be central in targeting freshly isolated myeloiddysplastic syndrome (MDS) blasts [56]. Importantly, the blockade of inhibitory KIR and CD94/NKG2A increase NK cell mediated killing of leukemic blasts indicating that they express enough HLA-class I to at least partially inhibit NK cells [57]. On the other hand, experimental evidences suggest that the activating counterparts of HLA-class I specific receptors (such as KIR2DS1^+^), could play a role in in the eradication of AML blasts [58,59].

The importance of the NK cell anti-tumor activity in tumor surveillance is corroborated by many studies showings that NK cells are frequently suppressed/altered in hematologic malignancies.

In CML patients, low numbers of NK cells are associated with defects in their proliferation and weak NK cell cytolytic functions in comparison with healthy donor blood NK cells [60,61]. Similarly, in MDS, the cytolytic activity of NK cells is impaired, even in the presence of IL-2 stimulation in vitro, as compared to NK cells from healthy donors [62]. In these CML/MDS patients, PB NK cells display severe defects including down-regulation of activating receptors, reduced cytotoxic potential, and reduced cytokine-induced proliferation in vitro. Activating NK receptors, such as DNAM-1, NKp30 and NKp46, display a low expression on NK cell surfaces also in AML patients and a parallel increase of expression of CD94/NKG2A inhibitory receptor [63,64,65]. Along with the phenotypic defects, cytolytic activity and TNF-α and/or IFN-γ production are also impaired and associated with poor clinical outcomes [63,64,65,66]. 

Leukemic blasts may directly affect NK cell repertoire and functions. The continuous exposure to the cognate ligands expressed by AML blasts may favor the modulation of corresponding activating receptors on autologous NK cells, leading to an exhaustion of the NK-cell cytotoxicity [67,68]. Other markers such as CD96, expressed by a subset of NK cells, or CD200, expressed on AML, may suppress patients NK cell functions [69,70]. Similarly, increased proportions of NKp30^low^TIM-3^+^ NK cells were found in the PB of CLL patients [71] Moreover, leukemic blasts may release various soluble molecules, including soluble ligands of activating NK receptors, soluble factors, such as TGF-β or IL-10, reactive oxygen species (ROS), or express high levels of indoeamine-2,3-dioxygenase (IDO) and tryptophan catabolites, which inhibit NK cell functions and modulate NK cell receptors expression [72,73,74,75,76]. In AML patients’ serum the presence of soluble NKG2D-L (MICA, MICB and ULBP2) is associated with a down-regulation of the surface NKG2D expression leading to an impairment in NKG2D-mediated NK-cell activity [77]. Soluble ligands can be also detected bound to tumor-derived exosomes in patient’s serum. Exosomes from leukemia/lymphoma cells can express NKG2D-L leading to an inhibition of the NK cell activation [78]. On the other hand, while the NKp30 ligand soluble BAG6, released by lymphocytic leukemia cells, works as an inhibitory ligand of NKp30, exosome-bound BAG6 activates NK cells in chronic lymphocytic leukemia (CLL) [79]. 

Hematological malignancies may favor the expansion of selected NK cell subsets, inducing a perturbation of the NK cell repertoire: AML may induce in some patients a deep NK cell maturation blockade that correlated with poor patient outcomes [80]. A central issue is also whether leukemic cells, in particular leukemic stem cells (LSC) present in the bone marrow, may influence the survival of normal stem cells and their differentiation towards immune cells with potential anti-leukemia activity [81]. Leukemic blasts may contribute to generate aberrant BM niche that competes with normal ones and that favors leukemic stem cell clone survival. ALL blast may rebuild a permissive niche in response to chemotherapy [82] while aberrant BM mesenchymal stromal cells (α-SMA^+^ mesenchymal stem cells) and hypoxia may contribute to the reduction of NK cell cytotoxicity against autologous AML blasts [83]. AML can also interfere directly with NK cell precursors proliferation and functional maturation. We showed, in a model of in vitro NK cell differentiation, that IL-1β-releasing AML blasts could inhibit the recovery of CD34^+^-derived CD161^+^CD56^+^ cells, resulting in a reduced generation of ILC3 and NK cells [84]. Thus, it is possible that IL-1β released by residual AML blasts may alter the BM microenvironment and suppress the proliferation of NK cell precursors [85] Finally, AML blasts may induce the expression of the transcription factor aryl hydrocarbon receptor (AHR) that activates miR-29b in NK cell precursors impairing their maturation and functions [86].

## 4. NK Cells and Solid Tumors 

Malignant cells derived from many solid tumors often express the ligand(s) for one or more of the major activating NK receptors including NCRs, DNAM-1, and NKG2D. Beyond their cytotoxic function, NK cells, are found to recruit to tumor sites DCs able to effectively prime T-cell response. In human cancers, intra-tumoral CCL5, XCL1, and XCL2 transcripts closely correlate with NK cells/cDC1 gene signatures and are associated with increased overall patient survival in several cancer types [87]. In addition, these innate cells, through secretion of FlT3-L, control the abundance of intra-tumoral stimulatory DCs and their frequency, that directly correlates with survival of melanoma patients receiving anti-PD-1 therapy [88].

Although many studies have deeply characterized the anti-tumor activity of NK cells both in vitro and in animal models, the exploitation of these cytotoxic cells for the therapy of solid tumors is still far to achieve. These difficulties are essentially related to the few numbers of NK cells present in the tumor bed and the suppressive effects mediated by the local tumor microenvironment on the NK cell effector mechanisms.

Early immunohistochemical studies in tumor biopsies suggested that NK cells might be effective during antitumor responses since their detection correlated with good prognosis of cancer patients [89,90,91]. However, NK cells present in solid tumors (including lung, gastric, colorectal) are usually scarce. Few exceptions are represented by renal cell carcinoma (RCC) and gastrointestinal stromal tumors (GIST), which are infiltrated by a significant number of NKp46^+^ cells [92,93]. Of note, different from hematological malignancies, tumor-infiltrating (TI) NK cells are usually not in direct contact with neoplastic cells, but they are rather located within the stroma. 

Besides their relative frequency and numbers, a key limiting factor of tumor-killing capacity of NK cells is their functional impairment. Solid TI-NK cells often display a CD56^bright^ phenotype and/or a reduced expression of the activating receptors resulting in a de-potentiated anti-tumor activity. In breast cancer, NK-TILs are enriched in CD56^bright^ cells expressing NKp44 and CD25 and CD69 activation markers, but exhibit low levels of NKp30, NKG2D, DNAM-1 and CD16 and poor cytotoxic potential [94]. In early-stage NSCLC, NK-TILs are mostly CD56^dim^, but express limited amounts of activating receptors (NKp30, NKp80, CD16, and DNAM-1) and display a low degranulation and cytokine release potential [95]. In RCC, infiltrating NK cells express high levels of CD94/NKG2A inhibitory receptor contributing to decreased NK cell activity [92] while in GIST patients, NK cells express predominantly the immunosuppressive NKp30c isoform and display a CD56^bright^ CD16-KIR- phenotype [93,96]. As mentioned above, tumor infiltrating NK cells and circulating NK cells may upregulate several inhibitory checkpoint receptors. TIGIT was found to be associated with NK cells with exhausted phenotype in colon cancer patients [97]. TIM-3 is upregulated on NK cells in several types of cancer while increased proportions of CD56^+^CD96^+^ NK cells can be found in the intra-tumor tissues of HCC: their expression level is associated with poor clinical outcomes or advanced stage of diseases [98,99,100,101,102]. Several studies in humans have shown that NK cells from cancer patients express PD-1 like ovarian cancer and Kaposi sarcoma [103,104]. Of note, blockade of TIM-3, PD-1, TIGIT may restore NK cell cytotoxicity or IFN-γ production, suggesting that the therapy with immune-checkpoint inhibitors (ICI) may contribute to restore also NK cell function [36,105].

In order to gain further insight about the functional states of human NK cells in solid tumors, a recent study analyzed the transcriptome of TI-NK cells isolated from human melanoma metastases compared with circulating NK cells [106]. Single-cell RNA-seq analysis of TI-NK cells identified different NK cell populations with specialized gene expression programs. Some NK cell subsets are found to expressed high levels of XCL1 and XCL2 chemokines genes that are critical for cross-presenting XCR1+ DCs recruitment into tumors [87], whereas other subsets show a higher perforin and granzyme B expression. This analysis also reveals that TI-NK cells express higher levels of the *KLRC1* gene encoding NKG2A inhibitory receptor than circulating NK cells.

Other tumor-residing cells or tumor cells themselves can hamper NK cell function at the tumor site by mechanisms commonly exploited also by hematological tumors. Thus, tumor associated macrophages (TAM) and other immature myeloid cells (myeloid derived suppressor cells, MDSC) can polarize a Th2 response and/or produce suppressive factors such as IL-10, TGF-β, ROS or deplete intracellular L-arginine [107]. T-reg represent another cell subset those accumulation in tumors correlates with impaired immune function and poor prognosis. A T-reg increase and a low NK cell activity has been described in gastrointestinal stromal tumor (GIST) and hepatocarcinoma (HCC) tumor-bearing subjects [108]. Tumor-associated fibroblasts, (TAF) are considered to play a pivotal role in mediating suppressive activity toward NK cells. TAF derived from different solid tumors were shown to inhibit NK cell function through both cell-to-cell contact and release of PGE2, which abrogate the IL-2-induced up-regulation of NKp44, DNAM-1 and NKp30 [109,110,111]. 

Tumor cells can hamper NK immune response by inhibitory mechanisms such as indoleamine 2,3-dioxygenase (IDO) expression and/or PGE2 production in metastatic melanoma, modulating expression of NKp30, NKp44 and NKG2D [112]. Other soluble tumor-derived factors such as TGF-β, Macrophage migration inhibitory factor (MIF), MUC-16 and adenosine [113] can impair NK cells. In neuroblastoma, TGF-β inhibits NK cell functions by modulating activating receptor expression and chemokine-receptor repertoire, possibly interfering with their ability to migrate and accumulate into tumor nest [114]. MIF and MUC-16 glycoprotein, expressed in ovarian tumor, are able to down-regulate NKG2D and to interfere with the formation of the synapses between tumor and NK cells [115,116]. In addition, shedding of MIC-A (NKG2D ligand) or of BAT3/BAG6 and B7H6 (ligands of NKp30), is a tumor escape mechanisms commonly reported [117,118]. Finally, down-modulation of NK cell activity can also be mediated by inhibitory signals triggered by the engagement of NKp44 receptor with its ligand proliferating nuclear cell antigen (PCNA) expressed in different tumor types [119].

Hypoxia (a condition which often characterizes tumor tissues) can both favor the selection of tumor cells with increased invasive and metastatic potential and alter the phenotypic and functional features of tumor-infiltrating immune cells. Along this line, we have described that hypoxia can significantly alter both the expression and function of major activating NK receptors, with the remarkable exception of CD16, thus allowing NK cells to maintain their capability of mediating ADCC [120]. 

## 5. TKI—Targeted Therapy Effects on NK Cells in Hematological Malignancies and GIST

TKI have been the first compounds designed to exert TT and are now a consolidated treatment of Ph^+^CML and Ph^+^-acute lymphoblastic leukemia (Ph^+^ALL) [121,122]. Recently, the US Food and Drug Administration (FDA) approved gilteritinib (Xospata^®^, Astellas Pharma inc., Tokyo, Japan), an FMS-like TK3-internal tandem duplication (FLT3-ITD) inhibitor, for the treatment of relapse/refractory AML [123,124]. Ph^-^myeloproliferative neoplasms (MPN) often harbor gain-of-function mutations that result in a V617F amino acid change in the JAK2 protein, mediating constitutive activation of the JAK/STAT (signal transducer and activator of transcription) pathway [125,126]. Ruxolitinib (Jakavi,^®^, Novartis, Basilea, Switzerland) is an JAK inhibitor already approved for the treatment of myelofibrosis and polycythemia vera [126]. 

TKIs such as imatinib mesylate (Gleevec^®^ Novartis, Basilea, Switzerland), nilotinib (Tasigna^®^, c Novartis, Basilea, Switzerland), and dasatinib (Sprycel^®^ Bristol-Myers Squibb, New York, NY, USA) are used as the front-line therapy in Ph^+^CML and Ph^+^ALL; only Imatinib has also been approved for the first-line therapy of GIST, characterized by c-KIT and PDGFRA mutations [127,128], renal cell carcinoma (RCC) and epidermal growth factor receptor (EGFR) mutation-positive non small cell lung cancer (NSCLC) [129,130]. TKIs inhibit the signal transduction pathways of aberrant tyrosin kinases p210 and p190 encoded by the aberrant fusion gene Breakpoint cluster region protein/Abelson murine leukemia viral oncogene homolog 1 (BCR/ABL) which favor blasts proliferation, apoptosis inhibition, and response to regulation factors [131].These drugs can inhibit several targets such as c-KIT, Platelet-derived growth factor (PDGF) but only dasatinib interferes also with ephrin receptor (EPH) and Src kinase that are overexpressed on tumor cells. In particular, dasatinib is capable of crossing the blood brain barrier and acts on brain metastases caused by Ph^+^LLA. Tyrosin kinase inhibitory drug therapy is not curative: some 50–60% of patients can develop resistance to the first generation imatinib and to the second generation TKI after imatinib failure. Ponatinib (Iclusig^®^, ARIAD Pharmaceuticals, Inc., Cambridge, MA, USA) a third generation TKI, has been designed for the treatment of Ph^+^ALL and Ph^+^CML with T315I mutation [132]. Ruxolitinib is supposed to also be not curative, but it leads to an excellent symptom control in MPN patients. However, therapeutic effects seem independent from mutational status suggesting a profound modification of inflammatory tumor microenvironment.

TT agents have been approved also for the treatment of CD20^+^ B lymphoid malignancies such as CLL and mantle cell lymphoma. In CLL, agents like the Bruton’s tyrosine kinase (BTK) inhibitor ibrutinib (Imbruvica^®^, Johnson & Johnson, New Brunswick, NJ, USA) and B-cell lymphoma 2 (Bcl-2) inhibitor venetoclax (Venclyxto^®^, AbbVie, North Chicago, IL, USA)are highly effective in CLL and induce deep remissions [133,134]. However, also these agents require a continuous treatment and may lose their efficacy upon acquisition of mutations by tumor cells [135,136].

**Figure 2:**
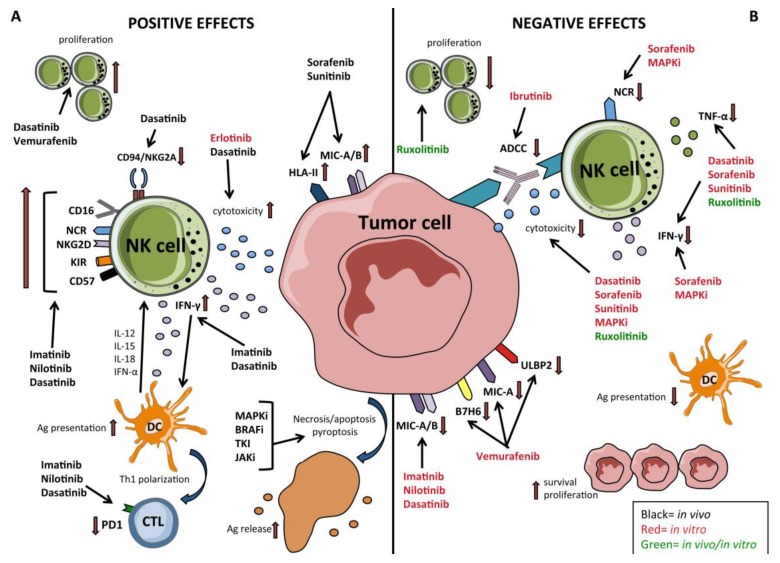
Effect of targeted therapies (TT) on tumor cells and Natural Killer (NK) cells **A**) The positive effects exerted by different TT on both tumor cells and NK cells. Apoptosis/necrosis/pyroptosis of tumor cells induced by TT results in release of tumor antigens for cross-presentation to DCs; In leukemia ibrutinib down-regulates the surface expression of the immune checkpoint ligand (PD-L1) of programmed cell death receptor-1 (PD-1); in leukemia imatinib, nilotinib and dasatinib upregulate the expression of NCR, NKG2D, CD16, and CD57 on NK cells while only dasatinib upregulates the amount of circulating NK cells and downregulates the surface expression of CD94/NKG2A inhibitory receptor on NK cells *in vivo*; dasatinib and imatinib enhance IFN-γ release while dasatinib also enhances NK cell cytotoxic activity. MEKi and BRAF inhibition of BRAF^V600E^ melanoma cells inhibit the secretion of immunosuppressive cytokines (VEGF, IL-6, and IL-10); in solid tumors sorafenib and sunitinib up-regulate the surface expression of HLA class II molecules and NKG2DL (MIC-A/B) *in vivo*. Erlotinib enhances NK cell cytotoxicity *in vitro*. Vemurafenib upregulates the amount of circulating NK cells *in vivo*. **B**) The “unwanted” effects exerted by different TT on both tumor cells and NK cells. In leukemia, imatinib, nilotinib and dasatinib downregulate the surface expression of NKG2DL (MIC-A/B) and upregulate the expression of KIRs on NK cells; in leukemia, ibrutinib exerts a negative effect on NK cell-mediated ADCC. In BRAF-mutated melanoma vemurafenib downregulates the expression of B7-H6 (NKp30L) and MIC-A (NKG2DL). MAPKi and sorafenib down-regulates NCR expression on NK cells; MAPKi, sorafenib, and sunitinib hamper NK cell function (cytoxicity, IFN γ -release); in leukemia; in MPN, ruxolitinib hinders NK cell proliferation and function (cytotoxicity and cytokine release).

Cumulative evidences indicated that the immune system would play a key role in the success of TKI therapy and in the control of emerging escape mutations that could lead to relapse. TKI therapy not only has a direct effect on onco-protein kinases but also has a key role in restoration of effector cells-mediated immune response such as NK cells, cytotoxic T lymphocytes (CTL) -CD8 and DCs (Figure 2). *In vitro* and *in vivo* studies revealed that TKI show the direct and indirect immunomodulatory effects involving natural killer (NK) cells, in particular in patients treated for CML or for GIST tumors [137,138,139]. On the other hand, the use of JAK inhibitors such as ruxolitinib showed a general dampening of immune response, also on NK cells, both in vitro and in vivo, offering an explanation of increase infections rate and of long term sides effects [140,141]. Regarding CLL, analyses of ibrutinib-mediated effects on NK cell proliferation and functions are limited: Kohrt and colleagues showed that ibrutinib inhibits NK cell proliferation, TNF-α production and lead to an anti-CD20-mediated ADCC impairment against chronic lymphocytic leukemia blasts mediated by rituximab [142]. 

In vitro analyses on the direct effects of imatinib, nilotinib and dasatinib reported conflicting results on NK cell proliferation and functions [137]. It has been described that the addition of Imatinib in co-cultures with NK cells and primary Chronic myeloid leukemia blasts didn’t impair NK cell cytotoxicity, while it was observed a significant reduction of IFN-γ production in the presence of nilotinib and dasatinib. In particular, dasatinib inhibited MAPK activation pathway involved in NK cell proliferation and activity. Moreover, it should take into account that TKI would favor leukemic blast escape from NK cell-mediated immunosurveillance because of they may induce surface modulation of MIC-A/B on leukemic blasts [143]. The bone marrow (BM) microenvironment plays a crucial role not only in favoring the resistance of leukemic stem cell (LSC) [144] to therapy but also in hematopoietic stem cell differentiation. It has been suggested that low dose of Imatinib has stimulatory effects on hematopoiesis [145], however, it is not clear whether chronic exposure to TKI may modulate cytotoxic lymphocytes maturation. In this context, we analyzed whether TKI treatment may influence NK cell differentiation and repertoire. The results suggested that dasatinib skews in vitro human CD56^+^ innate lymphoid cells differentiation from CD34^+^ hematopoietic stem cells towards non-cytotoxic Innate lymphoid cells type 3 (ILC3). The few NK cells undergoing in vitro differentiation in the presence of dasatinib express higher percentages of IFN-γ but had a reduced cytolytic activity against K562 leukemic cell line [146]. 

Despite these in vitro results, many clinical evidences suggest that TKI exert a positive immunomodulatory activity by suppressing and reducing myeloid-derived suppressor cells (MDSC) and T-reg and favoring the enrichment of cytotoxic lymphocytes [147,148]. The differences with in vitro data could be due to the in vivo short half-life of these compounds, that could reduce the potential negative off-sides effects. 

In vivo, TKI may differently affect NK cell repertoire [149]. Dasatinib has been shown to induce large granular lymphocytes (LGL) expansion that correlated with better CML prognosis. A rapid mobilization of NK cells and γ/δ T cells was observed during therapy, in particular immediately after a single oral drug dose [150]. Similarly, longitudinal analysis of BM-derived lymphocytes in patients undergoing TKI therapy (imatinib, nilotinib and dasatinib), revealed that early lymphocytes mobilization was associated with early molecular response [151]. 

Ex-vivo detailed phenotypic analyses on NK cell repertoire were lacking for long time. One report provided a short description of a comparative analysis among patients treated with different TKI [152]. Data indicated that imatinib-treated patients displayed on PB-NK cells an increased expression of the main activating receptors (NCR and NKG2D) while dasatinib patients showed an increased expression of KIR2DL1. Recently, a longitudinal study of 88 CML patients showed that dasatinib induced down-regulation of NKG2A on PB NK cells, leading to enhanced cytotoxicity and favoring CML patients’ clinical response. This effect wasn’t detectable in nilotinib- or imatinib- treated patients: in vitro experiments suggested that dasatinib inactivated p38 mitogen-activated protein kinase (MAPK) affecting nuclear distribution of GATA Binding Protein 3 (GATA3) which is involved in regulation of NKG2A transcription [153]. Finally, Hughes et al. demonstrated that CML patients in major molecular response (MMR) have an increase of CD56^+^CD94/NKG2C^+^NKG2D^+^NCR^+^KIR^+^ NK cells as compared to diagnosis, consistent with the restoration of a PB-NK cell repertoire typical of healthy individuals. Of note, authors observed a parallel decrease of MDSC, T-regs and of CD3^+^PD-1^+^ T cells confirming that molecular response require a global effect on immune cell repertoire restoration, thus suggesting the possibility that combined therapy that would include TKI and the use of immune check point inhibitors for CTLA-4 and/or PD-1 pathway might increase the number of patients achieving MMR [154].

All these evidences provide important clues to identify biomarkers that could help to define CML patients with better prognosis and eligible for a successful therapy discontinuation. Indeed, a high rate of CML patients achieve a deep molecular response (DMR, BCR-ABL ≤0.1%), but there are many concerns regarding the long-term sides effects of TKI therapy, and the economic burden of these therapies. Thus, one of the major goals is actually the possibility to induce a durable DMR (≥2 years) to allow a successful treatment free remission (TFR) [6,155,156]. In this context, the achievement of a rapid, durable and deep DMR seems to be crucial, thus, the contribution of immune system in this could play an important role [157,158]. Two reports suggested an increase of mature CD56^+^CD57^+^CD62L^−^ NK cell numbers in CML patients PB, that was associated with successful Imatinib discontinuation and deep molecular response in dasatinib treated patients [159,160]. In IMMUNOSTIM clinical trial, after imatinib treatment cessation, non-relapsing patients had a significantly increase of cytotoxic CD56^dim^, which was maintained over time, as compared to patients relapsing [161]. On the other hand, another study analyzed immune response in patients who, after DMR achievement with any TKI, underwent 2 years consolidation therapy with dasatinib and then discontinued treatment. Researchers found that patients who displayed higher percentages of PB-NK cells during TKI treatment had a higher DMR rate achievement. However, once started dasatinib consolidation treatment, a specific transient increase of circulating CD56^+^CD57^+^ NK cell percentages was unfavorable and associated to a rapid molecular relapse after therapy discontinuation [162]. The possible mechanisms explaining this phenomenon were not clear and a more detailed analyses of the PB or BM NK cells could have been be useful to address this issue.

Beyond the general positive effect that TKI therapy may directly exert in vivo on immune cell recovering and functional restoration, it is conceivable that patient’s genetic features may contribute to identify markers predictors for TKI therapy. It has been described a possible role of NK polymorphic patterns of KIR receptors to predict TFR in CML patients [163]. The absence of KIR2DS1, was associated with a better response to imatinib [164]. Yeung et al. observed that KIR2DL5B genotype could predict clinical outcome in CML-treated patients [165]. 

The clinical implication of TKI therapy in the treatment of solid tumor improved overall survival, reducing use of classical chemotherapy. Few studies in solid tumor regarding TKI modulation of NK response in vivo were performed, most of them on GIST patients. Imatinib achieves disease control in 80% of advanced KIT-expressing GIST, with median progression-free survival of 20–24 months [128]. In GIST patients treated with imatinib at 12 months, it has been observed an increase of IFN-γ production mediated by NKp30 and NKG2D receptor on NK cells. The IFN-γ production from circulating NK cells after 2 months of imatinib treatment could be correlated with long-term survival in GIST patients [166]. The prolonged treatment with imatinib lead to decrease of MIC-A/B on GIST and an increase of NKp46^+^ NK cells infiltration into tumor core associating with a reduced relapse [93]. Of note, also in these patients it has been possible to discriminate between an improved NK cell response associated with imatinib treatment and the genetic profile of patients NK cell repertoire that can be predictive of a more favorable clinical outcome. In particular, the different expression of NKp30 isoforms and the presence of NKp30 soluble ligands sB7-H6/BAG6, are predictive biomarkers of response to imatinib in GIST patients [167]. 

## 6. Targeted Therapies and Their Immunomodulatory Effects on NK Cells in Solid Tumors 

In the last 15 years, beyond the use of TKI such as imatinib in neuroblastoma and GIST patients, solid tumor treatment therapy took advantage by the identification of novel agents targeting oncoproteins frequently expressed in several malignancies. In this context, the MAPK cascade is perhaps the most important oncogenic driver of human cancers [3]. Approximately 50% of cutaneous melanomas harbor the oncogenic *BRAF ^V600^* mutation that constitutively activates the mitogen-activated protein kinase (MAPK)/ERK-signaling pathway [168]. BRAF-mutation has subsequently been identified as a driver mutation in several other cancers (colon cancer, thyroid cancer, and hairy cell leukemia) [168,169,170]. Constitutive activation of the MAPK signaling pathway is involved in tumor progression including evasion of apoptosis, unchecked cell replication, neoangiogenesis, tissue invasion, metastasis as well as escape from immune response [171]. In 2008, BRAF-targeted therapies for melanoma were tested in clinical trials demonstrating high response rates (more than 70%) [172] and a significant increase in survival when compared to dacarbazine that led to the FDA approval of vemurafenib (PLX4032, Zelboraf^®^,Plexxikon, Berkeley, CA, USA) in 2011 [173]. 

Although treatment with vemurafenib greatly improved the control of metastatic melanoma progression clinical responses were limited in time (less than 12 months) in most patients [174]. The emergence of resistance may reflect reactivation of MAPK pathway due to new mutations, BRAF amplification, or activation of alternative pathways involving MAPK and PI3K/Akt [175]. In view of these limitations, new protocols have been designed in which BRAF-targeted therapies have been associated with MEK inhibitors (MEK-i), such as trametinib [176] or cobimetinib [177]. However, again an important limitation these combination strategies is that patients still progress within a year [176]. 

Several other TT have been designed and approved for the treatment of different malignancies. Sunitinib (Sutent^®^, Pfizer, New York, NY, USA), sorafenib (Nexavar^®^,Bayer, Leverkusen, Germany)and alectinib (Alecensa^®^, Chugai Pharmaceutical, Tokyo, Japan) are involved in the inhibition of different kinases like vascular endothelial growth factor receptor (VEGFR), fibroblast growth factor receptors (FGFR), FLT3, proto-oncogene rearranged during transfection (RET). In particular, sunitinib was developed for the treatment of GIST, advanced metastatic renal cell carcinoma (MRCC) and primitive neuro-ectodermal tumor (pNET). Sorafenib, used for the treatment of HCC, inhibits serin/threonine kinases such as BRAF, CRAF BRAF ^V600E^ and ERK1/2, while alectinib was developed to treat anaplastic lymphoma kinase (ALK)-positive NSCLC. Gefitinib (Iressa^®^, AstraZeneca, London, United Kingdom) and erlotinib (Tarceva^®,^ AstraZeneca, London, United Kingdom) are similar drugs that inhibit EGFR in metastatic NSCLC with EGFR-TK activating mutation [3]. 

The increase of compounds available for TT therapy produced large number of studies however, the most detailed analyses have been performed on MAPK pathway activation which is an important therapeutic target, particularly in melanoma. Differently from hematological malignancies it is not clear whether the emergence of resistance could be related also to an immunomodulatory effect exerted on tumor immunosurveillance. Indeed, these inhibitors may exert bystander effects on certain immune cells that depend on MAPK for their activation and/or proliferation. TT would play a crucial role acting directly on the tumor by inducing its senescence, triggering the release of antigenic debris, thus favoring the acquisition by tumor cells of immunostimulatory properties and a more “permissive tumor microenvironment” that restore antitumor immune response, including that one of NK cells (Figure 2) [8]. On the other hand, they could exert an inhibitory effect acting on the lymphocyte activation pathways orchestrated by MAPK. Thus, it would be crucial to clarify these effects. However, while there are several in vitro data on the effect on immune cells and in particular on NK cells, data derived from ex-vivo analyses on patients undergoing BRAF-i/MEK-i TT are limited. 

Accumulating evidences indicated that inhibition of the MAPK pathway in melanoma cells may result in the block of the production of tumor-derived immunosuppressive or pro-angiogenetic factors including IL-6, IL-10 and VEGF that are essential for cancer immune evasion and growth [178]. Several studies in mice suggested an improvement of NK cell response against tumors during BRAF-i therapy [179,180]. In humans, we showed that PLX4032, a selective BRAF-i, has no inhibitory in vitro effect either on NK cell phenotype (i.e., activating NK receptor expression) proliferation in response to cytokines (including IL-2, IL-15, and IL-18) or on NK cell functions [181]. Furthermore, PLX4720 (a research analogue of vemurafenib) increased pERK1/2, CD69 expression and IFN-γ release in human NK cell cultures exposed to IL-2 and triggered via NKp30 [180]. Finally, it has been demonstrated that circulating NK cell numbers were even increased in vemurafenib-treated patients [182].

On the other hand, MAPKi, developed to block aberrantly activated pathways in melanoma cells, can have unexpected effects by concomitantly blocking the same pathway in TI-immune cells. Indeed, MEKi have been shown to have immunosuppressive properties on NK cells and to block NK-mediated antitumor immune response. MEK-i PD0325901 strongly inhibited the surface expression of the main activating receptors and the anti-tumor activity of freshly isolated NK cells cultured with IL-2 or IL-15 while no functional inhibition occurred in NK cells exposed to a combination of IL-15 and IL-18, suggesting that IL-15 and IL-18 cytokines could be utilized in combination with MEK inhibitors to rescue NK cell anti-tumor potential in vivo, while in IL-15/IL-18-untreated and in BRAFi-treated NK cells only a small fraction of cells expressed CD16, the large majority of IL-15/IL-18 MEKi-treated cells expressed of this marker, thus allowing MEKi-treated NK cells to preserve their capability of mediating antibody-dependent cell cytotoxicity (ADCC). 

Interestingly, BRAF/MEK inhibitors have no effect when added to NK cells pre-activated with IL-2 or IL-15, thus offering an important clue for the development of novel therapeutic strategies combining these kinase-targeted agents with adoptive NK cell therapy [181]. Data regarding other tyrosine kinase inhibitors are limited, but it seems that they may exert an inhibitory rather than stimulatory effect on NK cells. Sorafenib has been systemically utilized in the therapy of the advanced HCC. Although it leads to a significant block of tumor progression, it affects the function of NK cells including proliferation (due to the blocking of the PI3K/AKT pathway), production of effector molecules (such as perforin and granzyme B), cytokines (including TNF-α and IFN-γ) and NK-cell mediated cytotoxicity in response to tumor targets, due to impaired ERK phosphorylation [183]. Other kinase inhibitors such as those targeting JAK involved in the signaling cascade of cytokine receptors, may influence NK cells [184]. Despite the JAK/STAT pathway is an attractive target for breast cancer therapy due to its frequent activation in breast metatasis, a clinical trial evaluating JAK inhibitors (JAKi, ruxolitinib) in advanced breast cancer failed (NCT01562873). In vitro, ruxolitinib treatment blocked STAT phosphorylation and strongly decreased both proliferation of NK cells and the killing capability of NK cells against carcinoma cells, indicating that JAK/STAT pathway inhibition impairs the anti-tumor potential of NK cells.

In addition, TT may interfere with the NK/target interactions through modulation of NK ligands on cancer cells. In vitro treatment of human melanoma cell lines with vemurafenib downregulated the expression of NKp30L (B7H6) and NKG2DL (MICA and ULBPs) thus reducing their immunogenicity to NK cells [185,186]. By contrast erlotinib, an EGFR tyrosine kinase inhibitor widely used for the treatment of NSCLC patients, significantly modulates in vitro the phenotype of lung cancer cell lines toward a more epithelial ones and promotes tumor sensitivity to NK-mediated lysis by restoring susceptibility to caspase-dependent pathways [187]. 

Taken together these studies highlight the importance of evaluating the effect of TT on NK cell function and NK/tumor crosstalk to prevent potential harmful bystander effect.

## 7. Conclusions 

Targeted therapy can arrest tumor progression and induce a sharp regression in patients bearing specific mutations. In hematological malignancies, cumulative evidences indicate that TKI may induce durable response and support the hypothesis that this achievement correlates with the restoration of immune response that would counteract the emergence of mutations that could lead to resistance and relapse. On the other hand, data on solid tumors suggest that TT is not able to achieve a long-lasting remission. Moreover, in solid tumor, the effects of TT treatment on immune system still report conflicting results. Thus, additional treatment options are necessary and the combination with immunotherapy may represent the most suitable one. Targeted therapy may provide a favorable time interval in which stimulation of immune system by immunotherapy may play a complementary role and elicit an efficient tumor clearance in all patients in which TT alone is not able to induce the restoration of immune response.

Currently, there are several ongoing clinical trials regarding the combination of TT/TKI therapy and other therapies such as pharmacological manipulation of immune response (with type I IFN or lenalidomide), use of (ICI), monoclonal antibodies, vaccines and oncolytic virus that should boost immune response in CP_CML, MRCC, NSCLC, melanoma and other type of cancers [157,188,189]. These approaches would provide a durable response in larger cohorts of patients overcoming drug-induced resistance and improving clinical outcome. In CML, current knowledge of the effect of TKI on NK cell recovery in vivo and on the frequency of NK cells in long term TFR patients, prompted to design clinical trials that include the use of type I IFN with nilotinib to enhance immune modulation before TKI therapy discontinuation (NCT02001818), or lenalidomide in combination with different TKI (ACTRN 12615001169538) that specifically should increase NK cell antitumor response. Trials that include the use of ICI (Nivolumab, anti-PD-1) in combination with dasatinib for CML treatment (NCT02011945) or in combination with ibrutinib for CLL (NCT02420912) are also on going. In solid tumors the role of NK cells may appear less relevant, however their activation may support a first line of tumor control by cytolytic activity, IFN-γ and TNF-a production, and by stimulating Th1 response through DCs editing [190,191]. Several trials with therapy combination are ongoing: NCT01738139 involves patients with different advanced solid malignancies, including GIST, treated with a combination of Imatinib and Ipilimumab (anti-CTLA-4). Similarly, other trials are evaluating the efficacy of the combined use of TT agents (vemurafenib, cobimetinib, sunitinib, erlotinib and others) with ICI (pembrolizumab, anti-PD1, atezolizumab, anti-PDL-1, ipilimumab) in melanoma, NSCLC and RCC [188,189,192]. Several clinical trials exploiting the use of anti_TIGIT/TIM-3 blocking mAbs are also on going, either as monotherapy or mainly in combination with other anti-PD-1 or anti-PDL-1 mAbs while no one in combination with TT agents [193].

Most of these trials are already closed but the results are still under evaluation. However, the recent advances of TT and of immunotherapy on the control of neoplastic disease justify the expectation of promising results. In this context, NK cells may play a key role, representing both an important tool to exploit for the achievement a durable remission and an immunological biomarker, detectable in patients PB sample and tumor biopsies, that might work as prognostic factor of clinical outcome in different cancer patients.
